# Clinical Factors Affecting the Surgical Outcomes of Endoscopic Dacryocystorhinostomy

**DOI:** 10.3390/jcm15166212

**Published:** 2026-08-11

**Authors:** Min Seok Kim, Hahn Jin Jung, Soo Kyoung Park, Seung Hyuk Yang, Shin Hyuk Yoo, Ji-Hun Mo

**Affiliations:** 1 Department of Otorhinolaryngology, Dankook University College of Medicine, 201 Manghyang-ro, Dongnam-gu, Cheonan 31116, Republic of Korea; 2 Department of Otorhinolaryngology-Head and Neck Surgery, Chungbuk National University Hospital, Chungbuk National University College of Medicine, Cheongju 28644, Republic of Korea; hahnjin2@naver.com (H.J.J.);; 3 Department of Otorhinolaryngology-Head and Neck Surgery, Chungnam National University Sejong Hospital, Chungnam National University College of Medicine, Daejeon 35015, Republic of Korea

**Keywords:** dacryocystorhinostomy, nasolacrimal duct obstruction, endoscopic surgery, epiphora, allergic rhinitis

## Abstract

**Objectives:** Factors influencing outcomes of endoscopic dacryocystorhinostomy (DCR) for primary acquired nasolacrimal duct obstruction remain unclear. We evaluated clinical factors associated with surgical success. **Methods:** We retrospectively reviewed patients who underwent endoscopic DCR for this indication (2012–2022). Allergic rhinitis, obstruction level and type, canalicular intubation stent (CIS) insertion, and adjunctive procedures were analyzed against surgical success, epiphora scores, and SNOT-22 change using multivariate logistic regression. **Results:** Among 300 eyes (259 patients), the success rate was 82.0%. Epiphora scores improved from 7.6 ± 1.3 to 4.0 ± 2.3 and SNOT-22 from 17.3 ± 19.1 to 11.1 ± 14.2 (both *p* < 0.001). Sac-level and duct–sac junction obstructions had higher success than canalicular obstruction, and allergic rhinitis was associated with less epiphora improvement. CIS insertion was associated with lower success; however, this was confined to functional (42.0% vs. 92.0%) and canalicular-level (53.3% vs. 77.6%) obstruction—where CIS was selectively used—and absent in drainage-level obstruction (85.3% vs. 85.7%), indicating confounding by indication. **Conclusions:** Endoscopic DCR yields favorable outcomes with significant epiphora and quality-of-life improvement. Obstruction level, allergic rhinitis, and CIS insertion were associated with surgical outcomes; the CIS association, however, likely reflects confounding by indication rather than a direct adverse effect, as CIS was selectively used in functional and canalicular-level obstruction.

## 1. Introduction

Endoscopic dacryocystorhinostomy (DCR) has become a widely adopted standard surgical treatment for nasolacrimal duct obstruction (NLDO), offering success rates comparable to those of the conventional external approach while providing the additional benefits of avoiding skin incisions and facilitating faster postoperative recovery [1,2]. Advances in endoscopic instrumentation and surgical techniques have significantly improved surgical outcomes, making endoscopic DCR the preferred treatment modality in many tertiary and general hospital settings [2].

Despite these advancements, a proportion of patients still experience surgical failure. Previous studies have reported failure rates ranging from 5% to 20%, depending on the study population, surgical technique, and follow-up duration [3,4]. To improve surgical success, various adjunctive methods have been investigated, including silicone tube intubation, mucosal flap techniques, mitomycin C application, and simultaneous correction of anatomical abnormalities such as septal deviation or sinonasal disease [5].

Previous studies have identified several prognostic factors influencing surgical outcomes following endoscopic DCR [6,7]. Anatomical obstruction is generally associated with a more favorable prognosis than functional obstruction, which often lacks an identifiable mechanical cause. The level of obstruction—whether pre-sac, sac, or post-sac—has also been shown to significantly influence surgical success, with pre-sac or canalicular obstructions generally associated with poorer outcomes [8]. Additionally, patient-related factors, including advanced age, diabetes mellitus, and prior trauma or surgery, have been reported to negatively impact surgical success rates.

Furthermore, the necessity and optimal duration of silicone tube intubation remain topics of ongoing debate, with some studies supporting its routine use to maintain ductal patency, and others suggesting that it may be unnecessary in selected cases [9]. Allergic rhinitis has also been proposed as a potential prognostic factor. Several studies have suggested that chronic nasal inflammation associated with allergic rhinitis may impair wound healing or increase the risk of postoperative granulation tissue formation, thereby negatively impacting surgical outcomes [10]. Understanding the relative impact of these factors is essential for optimizing patient selection, tailoring surgical techniques, and improving long-term outcomes.

In this study, we retrospectively analyzed patients who underwent endoscopic DCR to identify prognostic factors associated with surgical success. By evaluating clinical variables such as demographic characteristics, obstruction type and level, surgical adjuncts, and comorbidities, we aimed to identify the factors that most significantly influence endoscopic DCR outcomes in a real-world clinical setting.

## 2. Materials and Methods

### 2.1. Study Design and Population

**Figure 1:**
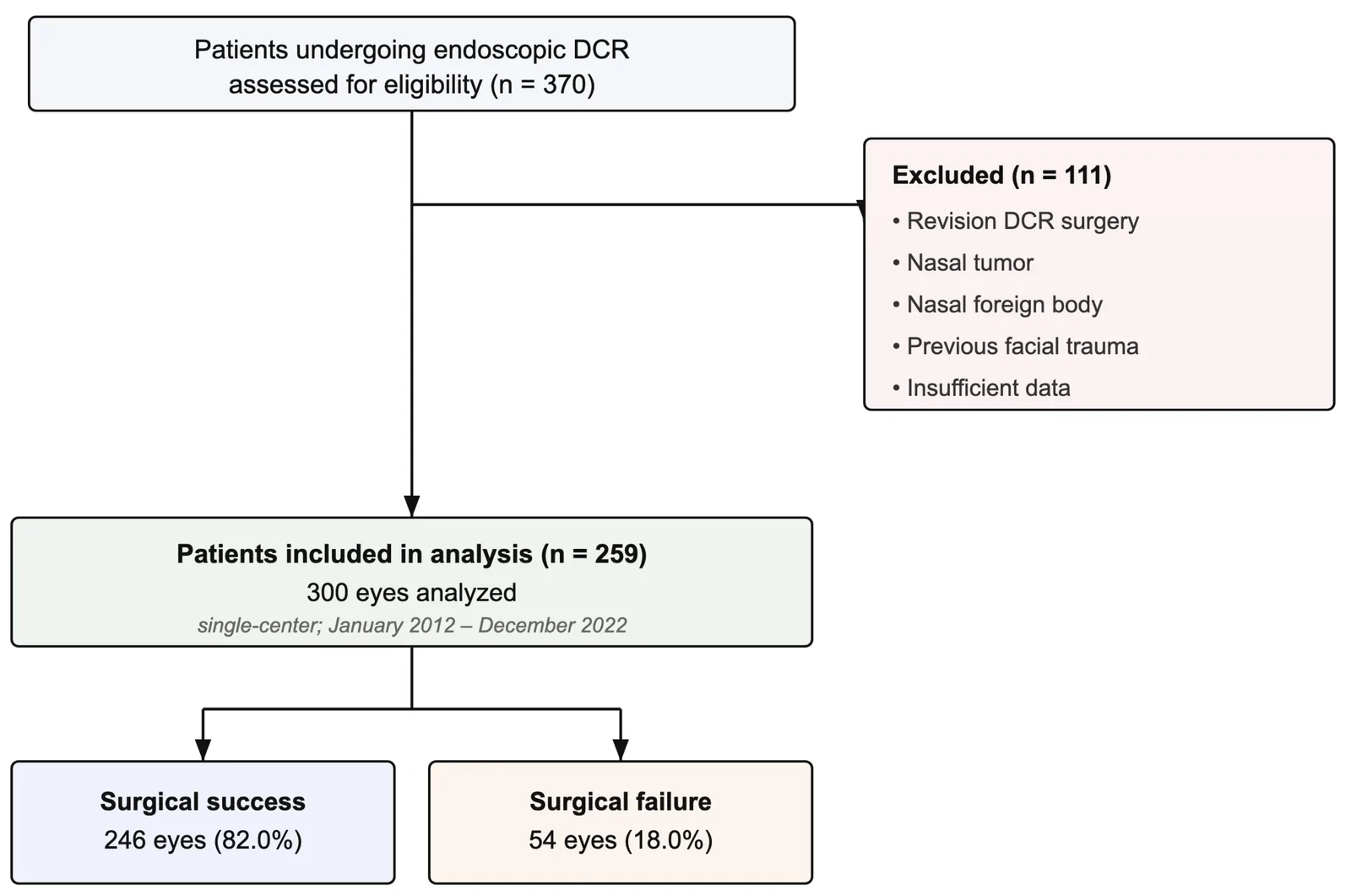
CONSORT-style flow of patient selection and surgical outcomes. DCR, dacryocystorhinostomy.

This single-center retrospective study reviewed the medical records of patients who underwent endoscopic DCR at Dankook University Hospital between January 2012 and December 2022. Care was provided by a multidisciplinary team (Departments of Otorhinolaryngology and Ophthalmology) within the same institution. The inclusion criteria were patients diagnosed with primary acquired NLDO who underwent endoscopic DCR and had complete clinical records. The exclusion criteria included revision surgery, nasal cavity tumors, the presence of a foreign body, previous facial trauma, and/or insufficient data for analysis. Insufficient data were defined as the absence of a preoperative or postoperative epiphora score, missing endoscopic follow-up for patency confirmation, or an undocumented obstruction level. A total of 259 patients (300 eyes) were enrolled after applying these criteria from an initial cohort of 370 patients (Figure 1).

### 2.2. Demographic and Clinical Variables

The following variables were recorded and analyzed: age, sex, past medical history (including diabetes mellitus and hypertension), and the presence of allergic rhinitis (diagnosed using ImmunoCAP, skin testing, or multiple allergen simultaneous tests (MAST). Additional variables included obstruction type (anatomical vs. functional), obstruction level (common canaliculus, lacrimal sac, duct–sac junction, and lacrimal duct), and whether adjunctive procedures such as endoscopic sinus surgery (ESS) or septoplasty were performed. The use of a canalicular intubation stent (CIS) and its duration of placement (typically 3–6 months) were also documented.

### 2.3. Allergic Tests

Allergy testing was performed using skin prick tests (SPTs), MAST, or ImmunoCAP^®^ assays, depending on patient age and clinical circumstances. SPTs were conducted using 50 common aeroallergens, including house dust mites, grasses, trees, weeds, feathers, cockroaches, cats, dogs, and molds. The tests were performed and interpreted by experienced personnel. For each allergen, the longest wheal diameter and its orthogonal diameter were measured, and the mean wheal diameter was calculated. A response was considered positive if the mean wheal diameter was equal to or greater than that of the histamine-positive control. All saline controls yielded negative results. Patients with at least one positive result were classified as allergic [11].

In patients who were too young to undergo SPTs or were taking medications that could interfere with skin test results, MAST or ImmunoCAP^®^ assays were performed. MAST was conducted using MAST pette chambers (MAST Immunosystems, Mountain View, CA, USA) containing 30 allergens. After incubation and washing, enzyme-tagged anti-IgE and a luminescent substrate were sequentially applied, and signals were measured using a MAST Optigen luminometer (Hitachi Chemical Diagnostics Inc., Mountain View, CA, USA). Results were classified into classes 0–4, with class ≥ 2 considered positive [12].

ImmunoCAP^®^ testing (Phadia AB, Uppsala, Sweden) was performed on blood samples collected during preoperative evaluations. Six allergen panels were tested: *Dermatophagoides pteronyssinus*, *Dermatophagoides farinae*, mold mix, tree mix, grass mix, and weed mix. For *D. pteronyssinus* and *D. farinae*, results were expressed in kUA/L and classified into classes 0–6, with class ≥ 2 considered positive. For the remaining allergens, results were reported as positive or negative. Patients with at least one positive result on either MAST or ImmunoCAP^®^ were classified as having allergic rhinitis.

### 2.4. Evaluation of Obstruction Type and Level

The type of obstruction was classified as either anatomical or functional based on intraoperative findings and lacrimal irrigation response. Anatomical obstruction was defined as a mechanically identifiable blockage detected during lacrimal probing or endoscopic examination, whereas functional obstruction was diagnosed when no apparent anatomical blockage was observed despite persistent symptoms and patent irrigation.

The level of obstruction was determined using lacrimal probing and intraoperative surgical exploration and was categorized into four levels: common canaliculus, lacrimal sac, duct–sac junction, and lacrimal duct. This classification was used to assess the impact of the obstruction site on surgical outcomes. The obstruction site was documented based on intraoperative localization of resistance or structural narrowing.

### 2.5. Surgical Procedures

All surgeries were performed under general anesthesia using a standard endoscopic DCR technique. After local decongestion of the nasal mucosa, a mucosal flap was elevated, and the frontal process of the maxilla and lacrimal bone were removed to expose the lacrimal sac. The medial wall of the lacrimal sac was tented using a lacrimal probe, and a vertical incision was made with a crescent knife. The lacrimal sac mucosa was marsupialized into the nasal cavity, and patency was confirmed by intraoperative lacrimal irrigation. A CIS was inserted selectively in eyes demonstrating severe nasolacrimal duct on dacryocystography and in eyes in which intraoperative probing of the lacrimal passage was difficult or incomplete—that is, cases with a tighter, more stenotic passage and a technically more demanding procedure. When used, the CIS was inserted through the canaliculi and clipped inside the nasal cavity for stenting, and was generally removed after 3 to 6 months. Adjunctive procedures, such as septoplasty or ESS, were performed simultaneously when indicated. Tissue adhesive was applied in selected cases to secure the flap. All procedures were performed by two expert, fellowship-trained otorhinolaryngologists at a tertiary referral center, using a single standardized protocol throughout the study period.

### 2.6. Outcome Evaluation

Surgical success was evaluated using both subjective and objective measures. Subjective improvement was assessed using a numeric rating scale (0–10) for epiphora severity, adapted from the Munk epiphora grading system [13], obtained via telephone interviews. Surgical success was defined a priori using combined criteria: resolution or improvement of epiphora on the 0–10 numeric rating scale together with endoscopic confirmation of a patent ostium at the last follow-up visit. A minimum postoperative follow-up of 3 months was required for outcome classification. Additionally, the 22-item Sinonasal Outcome Test (SNOT-22) was administered preoperatively and postoperatively.

### 2.7. The 22-Item Sinonasal Outcome Test Questionnaire

The SNOT-22 was administered preoperatively and postoperatively to assess changes in sinonasal symptoms and health-related quality of life. This validated questionnaire includes 22 items covering physical symptoms (e.g., nasal obstruction and facial pain), functional limitations (e.g., sleep disturbance), and emotional consequences (e.g., frustration and fatigue) [14]. Each item is scored on a Likert scale ranging from 0 (no problem) to 5 (worst possible problem), yielding a total score ranging from 0 to 110. Higher scores indicate greater symptom burden. Patients completed the questionnaire during preoperative evaluation and at their final follow-up visit. The change in total SNOT-22 score was used as an objective measure of postoperative symptom improvement.

### 2.8. Statistical Analysis

All statistical analyses were performed using SPSS version 25.0 (IBM Corp., Armonk, NY, USA). Categorical variables were compared using the chi-square or Fisher’s exact tests, as appropriate. Continuous variables were analyzed using the independent *t*-test or Mann–Whitney U test, depending on data distribution. Variables with *p* < 0.10 on univariate analysis, together with clinically established prognostic factors (age, obstruction level and type, CIS insertion, allergic rhinitis, and adjunctive procedures), were entered into a multivariate logistic regression model to identify independent predictors of surgical success. With 300 eyes and an 82.0% success rate (approximately 54 failures), the model accommodated about five covariates under the ten-events-per-variable rule. To address potential confounding by indication for CIS, we performed prespecified sensitivity analyses: (1) stratification of CIS-associated success rates by obstruction level, and (2) refitting of the multivariable model after restricting the cohort to eyes with lacrimal drainage-level obstruction (lacrimal sac, duct–sac junction, or lacrimal duct), thereby excluding functional and canalicular-level obstruction for which endoscopic DCR is not the primary indication. A *p*-value less than 0.05 was considered statistically significant.

## 3. Results

### 3.1. Demographics and Overall Outcomes

The mean age was 57.3 ± 13.5 years. Of the total cohort, 70 patients (27.1%) were male and 189 (72.9%) were female. Among these patients, 46 (17.1%) had diabetes mellitus, 89 (34.3%) had hypertension, and 44 (16.9%) had a history of allergic rhinitis. In addition, 47 patients underwent concurrent surgical procedures such as ESS or septoplasty (Table 1).

Surgical outcomes were assessed using three parameters: epiphora improvement score, change in SNOT-22 score, and surgical success rate. The mean postoperative epiphora score was 4.0 ± 2.3, which significantly improved from the preoperative score of 7.6 ± 1.3 (*p* < 0.001). The mean SNOT-22 score decreased from 17.3 ± 19.1 preoperatively to 11.1 ± 14.2 postoperatively (*p* < 0.001). The overall surgical success rate, defined by both subjective symptom improvement and endoscopic ostium patency, was 82.0% (246 of 300 eyes) (Table 2).

### 3.2. Age and Past Medical History

The presence of allergic rhinitis was associated with a slightly lower surgical success rate (76.7% vs. 81.0%), although this difference was not statistically significant. However, patients with allergic rhinitis demonstrated significantly less improvement in epiphora scores (*p* = 0.0117), whereas no significant difference was observed in SNOT-22 scores between the two groups (*p* = 0.1372) (Table 3).

No significant differences in surgical success rate or postoperative symptom improvement were observed according to age group or the presence of diabetes mellitus.

### 3.3. Obstruction Type and Level

**Figure 2:**
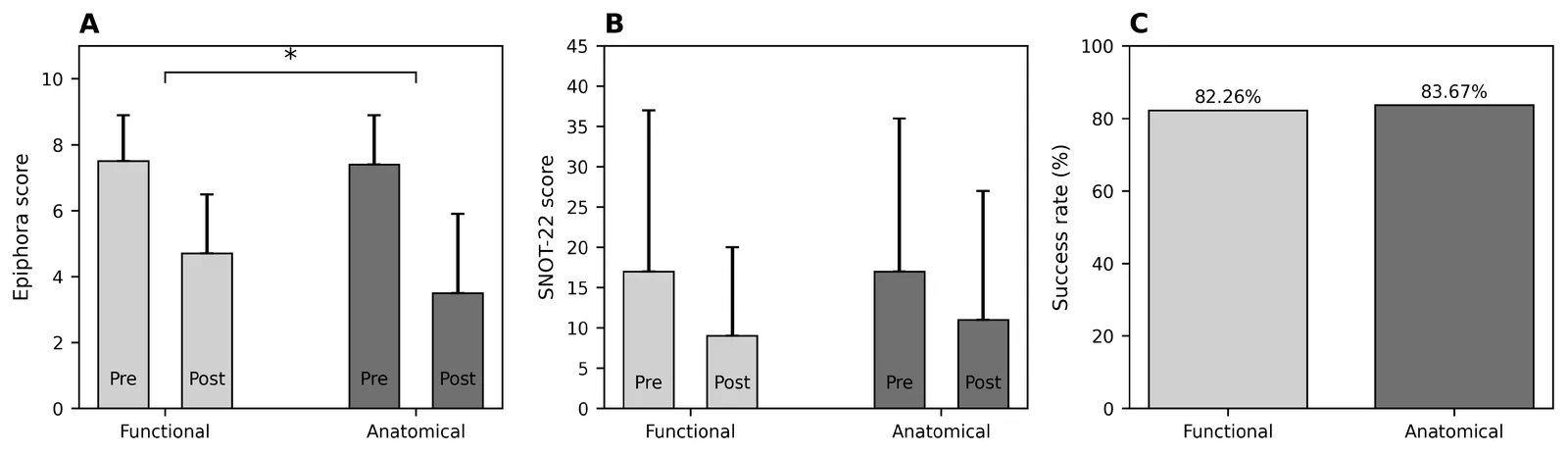
Comparison of surgical outcomes between anatomical and functional obstruction groups. Patients with anatomical obstruction showed significantly greater improvement in epiphora scores compared with those with functional obstruction (*p* = 0.0380) (**A**). No significant differences were observed in changes in SNOT-22 scores (*p* = 0.1614) (**B**) or surgical success rate (*p* = 0.712) (**C**). Error bars represent standard deviation. * means *p* < 0.05.

The overall surgical success rates were 83.67% and 82.26% in the anatomical and functional obstruction groups, respectively, with no statistically significant difference between groups (*p* = 0.712). However, patients with anatomical obstruction demonstrated significantly greater improvement in epiphora scores compared with those with functional obstruction (*p* = 0.0380). Changes in SNOT-22 scores did not differ significantly between the two groups (*p* = 0.1614) (Figure 2).

**Figure 3:**
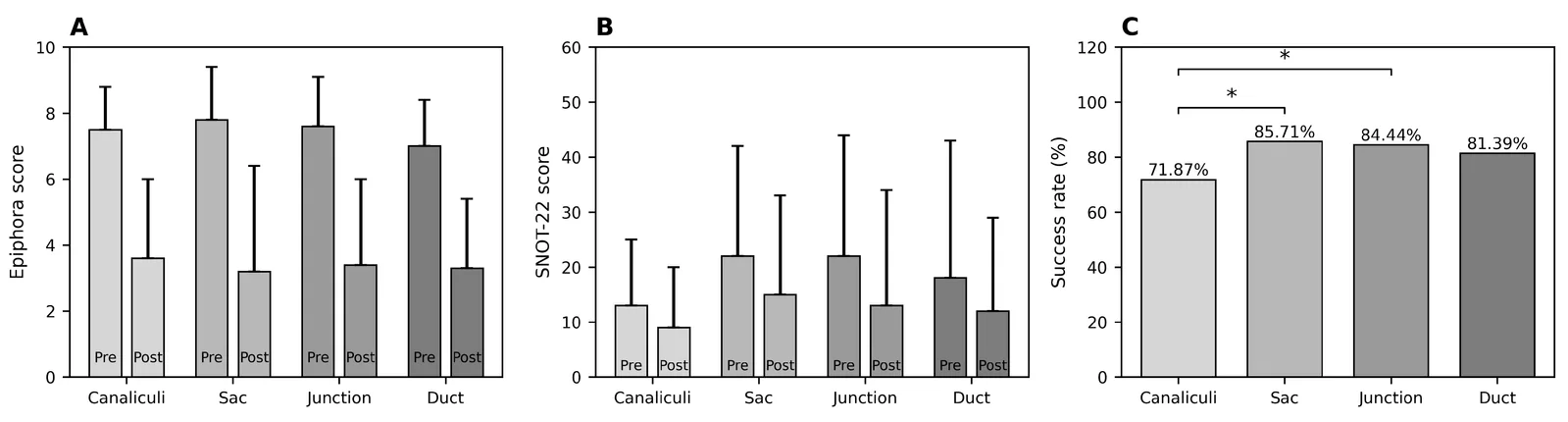
Surgical outcomes stratified by obstruction level. The success rate in the canaliculus-level group was significantly lower than that in the sac-level and junction-level groups (*p* = 0.0419 and *p* = 0.0334, respectively) (**C**). No significant differences were observed in changes in epiphora scores (*p* = 0.8431) (**A**) or SNOT-22 scores (*p* = 0.1955) (**B**) among obstruction levels. * means *p* < 0.05.

Surgical success rates varied according to the level of obstruction. Patients with lacrimal sac-level and duct–sac junction obstructions demonstrated higher success rates than those with common canaliculus or lacrimal duct obstruction. The success rate in the canaliculus-level group was significantly lower than that in the sac-level and junction-level groups (*p* = 0.0419 and *p* = 0.0334, respectively). In terms of symptom improvement, changes in epiphora and SNOT-22 scores did not differ significantly among obstruction level groups (*p* = 0.8431 and *p* = 0.1955, respectively) (Figure 3).

### 3.4. Canalicular Intubation Stent Tube Insertion

The surgical success rate was 76.5% in patients who underwent CIS tube insertion, which was significantly lower than the 86.6% observed in patients without stenting (*p* = 0.0323). Epiphora score improvement was also greater in the non-stented group (*p* = 0.0458). However, no statistically significant difference in SNOT-22 score change was observed between groups (*p* = 0.1467) (Table 4).

### 3.5. Adjunctive Surgery

Adjunctive procedures, such as septoplasty and ESS, were performed in a subset of patients. The surgical success rate was 87.5% in patients who underwent combined procedures and 81.2% in those who did not; however, this difference did not reach statistical significance. The change in epiphora scores did not differ significantly between groups (*p* = 0.6116). In contrast, the change in SNOT-22 score was significantly greater in the combined surgery group (*p* = 0.0287) (Table 5).

### 3.6. Multivariate Logistic Regression

Multivariate logistic regression analysis was performed to identify independent prognostic factors associated with surgical success (Table 6). The variables included in the model were age, sex, obstruction type, obstruction level, presence of diabetes mellitus, allergic rhinitis, CIS tube insertion, and adjunctive procedures. Among these, CIS tube insertion was identified as a significant negative predictor of surgical success (odds ratio [OR], 0.48; 95% confidence interval [CI], 0.25–0.92; *p* = 0.027). Obstruction type and allergic rhinitis were not significant predictors in the adjusted model (*p* = 0.356 and *p* = 0.185, respectively). In a prespecified sensitivity analysis, the lower success associated with CIS was confined to functional (42.0% vs. 92.0% in non-stented eyes) and canalicular-level (53.3% vs. 77.6%) obstruction. Among eyes with lacrimal drainage-level obstruction (lacrimal sac, duct–sac junction, or lacrimal duct), the surgical success rate did not differ by CIS status (85.3% vs. 85.7%; *p* = 0.94), and CIS was no longer associated with success when the multivariable model was restricted to these eyes (adjusted OR, 0.78; 95% CI, 0.28–2.19; *p* = 0.64).

## 4. Discussion

In this study, we investigated prognostic factors associated with surgical success following endoscopic DCR. The overall success rate was 82.0%, with significant postoperative improvements observed in both epiphora and SNOT-22 scores. Among the variables evaluated, obstruction level, CIS tube use, and allergic rhinitis were identified as factors associated with surgical outcomes.

Several previous studies have investigated prognostic factors in endoscopic DCR to improve surgical outcomes and minimize postoperative failure [6,7,8,9,10]. Various factors have been studied, including anatomical versus functional obstruction, the level of nasolacrimal duct blockage, silicone stent use, and patient-specific comorbidities such as diabetes mellitus and allergic rhinitis. Additionally, surgical refinements and adjunctive procedures, including ESS and septoplasty, have been explored to improve surgical outcomes. In this study, the overall surgical success rate was 82.0%, with significant postoperative improvements observed in both epiphora and SNOT-22 scores. This success rate is comparable to previously reported ranges of 80–95% for endoscopic DCR [1,2], reaffirming the procedure’s efficacy in treating primary acquired NLDO.

Regarding obstruction type, although anatomical obstruction showed a marginally higher surgical success rate and greater improvement in epiphora symptoms, the difference in success rate compared with functional obstruction was not statistically significant. This finding is consistent with previous studies reporting no significant differences in surgical outcomes between anatomical and functional NLDO [15]. However, some studies have suggested that functional blockages may be associated with poorer outcomes due to the absence of visible anatomical abnormalities that can be surgically corrected, potentially complicating intraoperative management and postoperative patency [16,17].

Allergic rhinitis was also associated with diminished improvement in epiphora symptoms, although it did not significantly affect overall surgical success. Previous studies evaluating the impact of allergic rhinitis on DCR outcomes are limited; however, existing evidence suggests that the chronic inflammatory state of the nasal mucosa may impair wound healing, promote granulation tissue formation, or compromise long-term patency of the ostium [18]. Pathological analysis has demonstrated that persistent allergic inflammation can induce mucosal hyperplasia and osteitic remodeling, potentially increasing the risk of restenosis [19]. In this study, patients with allergic rhinitis showed significantly less improvement in symptom scores, reinforcing the possibility that allergic rhinitis may negatively influence the postoperative healing environment. Although allergic rhinitis was not identified as an independent predictor of surgical success in the multivariate analysis, these findings suggest potential clinical relevance and underscore the need for further prospective studies to clarify its role in DCR outcomes. From a management standpoint, preoperative optimization of allergic rhinitis—using intranasal corticosteroids, antihistamines, and, where appropriate, allergen immunotherapy—may reduce perioperative mucosal inflammation and granulation and could help mitigate its negative influence on epiphora outcomes.

Obstruction level was identified as a significant factor influencing surgical outcomes in this study. Patients with lacrimal sac-level or duct–sac junction obstructions demonstrated higher surgical success rates than those with canaliculus-level obstruction. This finding is consistent with previous reports showing lower surgical success rates in canaliculus-level obstructions due to the greater technical difficulty of achieving adequate marsupialization and the increased risk of postoperative stenosis [8]. In contrast, more distal obstructions, particularly those involving the lacrimal sac, are generally more amenable to surgical correction through an endoscopic approach. As DCR is not the procedure of choice for canalicular-level obstruction, the lower success rate observed in this subgroup is an expected finding.

CIS tube insertion, although commonly performed to maintain ostium patency, was associated with lower surgical success rates and reduced symptom improvement in the overall cohort. Prior studies have reported inconsistent findings regarding the role of silicone stenting [20,21]. Some authors have argued that stenting may be unnecessary in uncomplicated primary DCR cases and may induce local inflammation, granulation tissue formation, and subsequent restenosis [21]. Conversely, other studies have suggested that stenting may facilitate wound stabilization and maintenance of ostium patency, particularly in patients with narrow anatomical configurations or at an elevated risk of postoperative closure [9]. In this cohort, patients who underwent CIS insertion experienced lower success rates and reduced improvement in symptom scores; although stent-related mucosal trauma or interference with mucosal apposition during healing is conceivable, this pattern is more likely explained by confounding by indication, as detailed below. These findings support the view that routine stenting should be reconsidered and that patient selection for CIS insertion should be individualized based on intraoperative assessment and risk stratification. Importantly, because CIS was applied selectively to eyes with nasolacrimal duct stenosis on dacryocystography and difficult intraoperative probing—both markers of a more challenging passage and poorer intrinsic prognosis—this negative association most likely reflects confounding by indication rather than a direct detrimental effect of the stent. Consistent with this interpretation, in a level-stratified sensitivity analysis the association was confined to functional and canalicular-level obstruction and disappeared among lacrimal drainage-level obstructions, where the success rate did not differ by CIS status (85.3% vs. 85.7%; *p* = 0.94), in keeping with reports that intubation may benefit anatomically challenging cases such as those with a small lacrimal sac [22]. Accordingly, the association should be interpreted with caution and not as a causal effect.

Adjunctive procedures such as septoplasty or ESS did not significantly alter the surgical success rate; however, patients in this group experienced greater improvement in SNOT-22 scores. This suggests that addressing coexisting sinonasal pathology may contribute to broader symptomatic relief, even if it does not directly influence lacrimal drainage patency. This interpretation warrants caution, however, as the need for concurrent septoplasty or ESS itself reflects coexisting nasal pathology; any associated difference in outcome may therefore be attributable to the underlying nasal disease rather than to the DCR or the adjunctive procedure per se.

This study has several limitations. The retrospective design may have introduced selection bias and limited causal inference. Subjective outcome measures, including epiphora scoring and telephone-based follow-up, may be subject to recall bias. Furthermore, certain diagnostic assessments, such as nasal endoscopy grading, allergy test standardization, and ostium size quantification, were not uniformly applied. Additionally, the decision to perform adjunctive procedures or insert a CIS tube was made at the surgeon’s discretion based on intraoperative findings, and the underlying rationale for these interventions was not standardized or systematically analyzed in this study. In particular, because CIS insertion and adjunctive procedures were indicated by disease-related features (nasolacrimal duct stenosis, difficult intraoperative probing, and coexisting nasal pathology), the associations of these interventions with outcome are subject to confounding by indication, and residual confounding cannot be fully excluded despite multivariable adjustment and sensitivity analysis. Furthermore, although based on the Munk grading system [13], the expanded 0–10 epiphora numeric rating scale used here has not itself been formally validated, and SNOT-22 is a sinonasal rather than a lacrimal-specific measure; validated lacrimal-specific tools such as the Lacrimal Symptom Questionnaire (Lac-Q) [23] are recommended for future studies. Finally, all surgeries were performed at a single institution by two surgeons over an extended study period; although a standardized protocol was used, inter-surgeon and temporal variability cannot be fully excluded, and the single-center design may limit the generalizability of our findings.

## 5. Conclusions

This study demonstrated an 82.0% surgical success rate for endoscopic DCR, with significant improvements in epiphora and SNOT-22 scores. Prognostic factors, including obstruction level, CIS tube insertion, and allergic rhinitis, were found to influence outcomes. The association between CIS tube insertion and lower success appears to reflect confounding by indication—CIS having been selectively used in functional and canalicular-level obstruction—rather than a direct adverse effect of the stent. These findings underscore the importance of thorough preoperative assessment and individualized surgical planning to optimize clinical outcomes in patients undergoing endoscopic DCR.

## Figures and Tables

**Table 1:** Demographic and clinical characteristics of patients who underwent endoscopic dacryocystorhinostomy (*n* = 259). Data are presented as number (%) or mean ± standard deviation (SD). Age was stratified into four categories. Comorbidities include diabetes mellitus (DM), hypertension (HTN), and allergic rhinitis (Allergy). Co-op indicates concurrent surgical procedures performed during DCR, including septoplasty and endoscopic sinus surgery (ESS).

Characteristics	Total (*n* = 259)
**Sex**	
Male: Female	70 (27.1%): 189 (72.9%)
**Age (y), mean ± SD**	57.3 ± 13.5
~49	79 (30.5%)
50~59	82 (31.6%)
60~69	58 (22.3%)
70~	40 (15.4%)
**DM**	46 (17.1%)
**HTN**	89 (34.3%)
**Allergy**	44 (16.9%)
**Co-op**	
Septoplasty	30 (11.5%)
ESS	17 (6.5%)

**Table 2:** Preoperative and postoperative symptom scores.

Characteristics	Mean Symptom Score
**Epiphora symptom score**	
Preoperative	7.6 ± 1.3
Postoperative	4.0 ± 2.3
**Total SNOT-22 score**	
Preoperative	17.3 ± 19.1
Postoperative	11.1 ± 14.2

**Table 3:** Surgical outcomes according to the presence of allergic rhinitis.

Outcome	Allergic Rhinitis (+)	Allergic Rhinitis (−)	*p* Value
Surgical success rate (%)	76.67	81.03	0.7811
Epiphora score improvement	3.09	4.64	**0.0117**
SNOT-22 score change	5.98	8.07	0.1372

**Table 4:** Surgical outcomes according to CIS tube insertion.

Outcome	CIS Tube (+)	CIS Tube (−)	*p* Value
Surgical success rate (%)	76.5	86.6	0.0323
Epiphora score improvement	2.769	3.822	0.0458
SNOT-22 score change	4.690	7.941	0.1467

**Table 5:** Surgical outcomes according to the adjunctive procedures (septoplasty and/or ESS).

Outcome	Combined Surgery (+)	Combined Surgery (−)	*p* Value
Surgical success rate (%)	87.5	81.2	0.3326
Epiphora score change	3.789	3.488	0.6116
SNOT-22 score change	9.875	5.099	**0.0287**

**Table 6:** Multivariate logistic regression analysis of factors associated with surgical success after endoscopic dacryocystorhinostomy. Independent variables included in the model were age, sex, obstruction type and level, presence of diabetes mellitus (DM), hypertension (HTN), allergic rhinitis, canaliculus intubation stent (CIS) tube insertion, and adjunctive procedures (endoscopic sinus surgery [ESS] or septoplasty). Odds ratios (OR) with 95% confidence intervals (CI) and *p*-values are presented.

	B	SE	Wald	*p*-Value	OR	95% CI	
DM	0.065	B	0.025	0.875	1.067	0.473	2.407
HTN	0.061	0.337	0.337	0.856	0.941	0.486	1.820
**CIS tube**	**0.679**	**0.308**	**0.308**	**0.027**	**1.972**	**1.078**	**3.605**
ESS	0.707	0.765	0.854	0.355	2.028	0.565	9.082
Septoplasty	0.423	0.508	0.695	0.404	1.527	0.453	4.129
Anatomical	0.022	0.373	0.004	0.953	1.022	0.492	2.123
Allergy	0.089	0.454	0.038	0.845	1.093	0.449	2.659

## Data Availability

The original contributions presented in this study are included in the article. Further inquiries can be directed to the corresponding authors.
